# Neurological signs in 23 dogs with suspected rostral cerebellar ischaemic stroke

**DOI:** 10.1186/s13028-016-0219-2

**Published:** 2016-06-07

**Authors:** Barbara Thomsen, Laurent Garosi, Geoff Skerritt, Clare Rusbridge, Tim Sparrow, Mette Berendt, Hanne Gredal

**Affiliations:** 1 Department of Veterinary Clinical and Animal Sciences, University Hospital for Companion Animals, University of Copenhagen, Dyrlægevej 16, 1870 Frederiksberg C, Denmark; 2 Davies Veterinary Specialists, Manor Farm Business Park, Higham Gobion, Hitchin, England, SG5 3HR UK; 3 Chestergates Veterinary Referral Hospital, Units E & F, Telford Court, Chestergates Road, Chester, Cheshire, England, CH1 6LT UK; 4 Fitzpatrick Referrals, Halfway Lane, Eashing, Godalming, Surrey, England, GU7 2QQ UK; 5 School of Veterinary Medicine, Faculty of Health and Medical Sciences, University of Surrey, Guildford, Surrey, England, GU2 7TE UK; 6 Stone Lion Veterinary Hospital, 41 High Street, Wimbledon, London, SW19 5AU UK

**Keywords:** Canine, Cerebellum, Cerebrovascular accident, Infarct, Ischemic, Occlusion, Rostral cerebellar artery, Syndrome, Vestibular

## Abstract

**Background:**

In dogs with ischaemic stroke, a very common site of infarction is the cerebellum. The aim of this study was to characterise neurological signs in relation to infarct topography in dogs with suspected cerebellar ischaemic stroke and to report short-term outcome confined to the hospitalisation period. A retrospective multicentre study of dogs with suspected cerebellar ischaemic stroke examined from 2010–2015 at five veterinary referral hospitals was performed. Findings from clinical, neurological, and paraclinical investigations including magnetic resonance imaging were assessed.

**Results:**

Twenty-three dogs, 13 females and 10 males with a median age of 8 years and 8 months, were included in the study. The Cavalier King Charles Spaniel (*n* = 9) was a commonly represented breed. All ischaemic strokes were located to the vascular territory of the rostral cerebellar artery including four extensive and 19 limited occlusions. The most prominent neurological deficits were gait abnormalities (ataxia with hypermetria *n* = 11, ataxia without hypermetria *n* = 4, non-ambulatory *n* = 6), head tilt (*n* = 13), nystagmus (*n* = 8), decreased menace response (*n* = 7), postural reaction deficits (*n* = 7), and proprioceptive deficits (*n* = 5). Neurological signs appeared irrespective of the infarct being classified as extensive or limited. All dogs survived and were discharged within 1–10 days of hospitalisation.

**Conclusions:**

Dogs affected by rostral cerebellar ischaemic stroke typically present with a collection of neurological deficits characterised by ataxia, head tilt, and nystagmus irrespective of the specific cerebellar infarct topography. In dogs with peracute to acute onset of these neurological deficits, cerebellar ischaemic stroke should be considered an important differential diagnosis, and neuroimaging investigations are indicated. Although dogs are often severely compromised at presentation, short-term prognosis is excellent and rapid clinical improvement may be observed within the first week following the ischaemic stroke.

## Background

In dogs affected by ischaemic stroke, one of the most common sites of infarction relate to the cerebellum. The blood supply to the cerebellum is ensured by two paired arteries; the rostral cerebellar artery (RCeA), usually originating from the caudal communicating artery (caudal part of the circle of Willis), and the caudal cerebellar artery (CCeA), originating from the basilar artery. The origin and formation of these vessels are, however, subject to large biological variation [1–3].

Ischaemic stroke is caused by a thrombotic or thromboembolic event, resulting in infarction with loss of neuro-function of the related vascular territory. Neurological signs accordingly reflect infarct topography. The clinical signs are usually sudden, non-progressive, and a gradual improvement is typically seen, although further neurological deterioration may develop within minutes to hours, or rarely up to days, of the acute event due to progressive cell death and a growing brain oedema [4–6].

In dogs, ischaemic stroke is increasingly recognised as a cause of acute neurological deficits. However, the number of publications is still limited, and only a few studies between 2005 and 2014 include a reasonable number of cases (ranging from 12 to 40 dogs) [4, 7–12]. Based on the current literature, it appears that infarcts located to the cerebellum may be particularly frequent in dogs [7, 9, 10, 13–17].

The majority of the reported cases of cerebellar stroke in dogs are related to the area of the RCeA [7, 9, 10, 16, 18–20], whereas CCeA related events seem rare [10, 21].

One of the cerebellum’s main functions is to modulate body movement and maintain equilibrium, the latter primarily via the flocculonodular lobe’s relation to the vestibular nuclei of the brainstem [22, 23]. These functions may thus be affected with a cerebellar stroke. In humans, the collection of symptoms that occurs with cerebellar infarcts may be referred to as the cerebellar stroke syndrome, which is characterised by vertigo, ataxia, nystagmus, and headache [24–26]. As strokes in dogs and humans compare in many aspects [10], a similar stroke syndrome in dogs might be expected. The possible identification and characterisation of a cerebellar stroke syndrome in dogs might promote the clinician’s suspicion of a stroke, which according to previous studies carries a fair to good prognosis, despite a severe clinical onset [4, 6]. This might reduce the risk of any premature decision-making towards euthanasia.

Although previously reported in dogs, new studies on cerebellar stroke are important, not only to confirm previous findings, but also to advance the current understanding of symptomatology and prognosis. The aim of this study was to further characterise neurological signs in relation to infarct topography in dogs with suspected cerebellar ischaemic stroke. Furthermore, short-term outcome for affected dogs was investigated.

## Methods

### Study design

A retrospective multicentre study was performed, including dogs examined from 2010–2015 at five veterinary referral hospitals (Davies Veterinary Specialists, Herts; Chestergates Veterinary Referral Hospital, Chester; Fitzpatrick Referrals, Surrey; Stone Lion Veterinary Hospital, London, and the University Hospital for Companion Animals, Copenhagen). Dogs with magnetic resonance imaging (MRI) changes suggestive of a cerebellar ischaemic stroke were eligible for inclusion.

Inclusion criteria were: a full medical record including history, physical and neurological examination, complete blood count, and serum biochemistry. Results of the cerebrospinal fluid analysis were included, when available. Exclusion criteria were: progression of neurological deficits beyond 24 h, any presence of concurrent non-cerebellar strokes, and treatment with immunosuppressive doses of corticosteroids.

All neurological examinations were performed by a DipECVN, a resident working under the supervision of a DipECVN, or by a DVM with a PhD in veterinary neurology.

The study was approved by the Local Administrative and Ethics Committee, Department of Veterinary Clinical and Animal Sciences, Faculty of Health Sciences, University of Copenhagen (Permission no. 1 N/2013).

### Diagnostic imaging

The following MRI equipment was used: 0.4T (Aperto Permanent Magnet, Hitachi) at Davies Veterinary Specialists (*n* = 10), 0.35T MRI (Vet-MR Grande, Esaote) at Chestergates Veterinary Referral Hospital (*n* = 7), 1.5T MRI (MAGNETOM Symphony, Siemens Healthcare) at Fitzpatrick Referrals (*n* = 3), and 0.2 T MRI (Vet-MR, Esaote) at Stone Lion Veterinary Hospital (*n* = 2) and the University Hospital for Companion Animals (*n* = 1). Dogs were placed in sternal or dorsal recumbency and scanned under general anaesthesia.

The images were obtained by using, at a minimum, transverse and sagittal T1-weighted (T1W), pre- and postcontrast, and T2-weighted (T2W) images.

Gadolinium dimeglumine (Magnevist, Schering^)^ at a dose of 0.1 mmol/kg IV, gadoteridol (Prohance, Bracco Imaging) at a dose of 0.1 mmol/kg IV, or Gadoteric acid (Dotarem, Guerbet) at a dose of 0.1 mmol/kg IV was used as the paramagnetic contrast medium. Due to the retrospective study setup, fluid attenuated inversion recovery, gradient echo, diffusion weighted imaging or angiography sequences were not available in all cases.

MRI changes were considered compatible with cerebellar ischaemic stroke if a well-defined lesion was seen within the vascular territory of either of the paired RCeA or CCeA and characterised by iso- or hypointense changes in T1W sequences, and hyperintensity in T2W sequences, and with no or little contrast enhancement and no or minimal mass effect.

A DipECVDI or DipACVR and a DipECVN evaluated the MRI scans. The first (BT) and last author (HG) performed the topographical classification. Both were blinded to the specific neurological signs of the dogs.

### Infarct topography

Topography of infarcts was classified according to the distribution area of either the RCeA or the CCeA. Lesions were localized to the right, to the left, or to the midline. Occlusions were defined as ‘extensive’ when the entire supply area of either the RCeA or CCeA was affected, and ‘limited’ when the affected area represented either a smaller cortical part of the supply area of the RCeA, i.e. the limited area of their arterial branches, or a perforating artery, i.e. lacunar infarcts located in the deep structures of the cerebellum. This classification was based on a subjective evaluation of the infarction visualized on MRI. Further classification of the infarcts into specific distal branches could not be determined due to inconsistency between selected MRI planes and sequences, given the retrospective nature of the data.

### Short-term outcome

Short-term outcome was confined to the hospitalisation period and classified as ‘good’ if dogs with suspected cerebellar ischaemic stroke improved to a stage where they could successfully be discharged and as ‘poor’ if they either died or were euthanized during the hospitalisation period. Duration of the hospitalisation time was registered according to extensive vs. limited occlusions.

### Statistical analysis

The statistics of the present study were mainly descriptive. For the comparison of mean ages between groups a *t* test was performed on normally distributed data using GraphPad Prism 6 (GraphPad Software, Inc. 7825 Fay Avenue, Suite 230 La Jolla, CA 92037 USA). Results are stated with ±standard error of the mean. *P* values ≤ 0.05 were considered statistically significant.

## Results

Twenty-three dogs were included in the study (ten from Davies Veterinary Specialists, seven from Chestergates Veterinary Referral Hospital, three from Fitzpatrick Referrals, two from Stone Lion Veterinary Hospital, and one from the Copenhagen University Hospital for Companion Animals). All dogs were referral cases, and the study group encompassed thirteen females (11 neutered) and 10 males (five neutered) with a median age of 8 years and 8 months (range 3–12 years). Breeds comprised the Cavalier King Charles Spaniel (*n* = 9), Greyhound (*n* = 2), Labrador Retriever (*n* = 2), Basset Hound, Cairn Terrier, English Cocker Spaniel, German Shepherd, Lurcher, Pointer, Shih Tzu, Tibetan Terrier, Weimaraner, and one medium-sized mixed-breed (*n* = 1). The Cavalier King Charles Spaniel was significantly younger at stroke onset (mean age 7 years ± 9 months) compared to non-Cavalier King Charles Spaniels (mean age 9 years and 1 month ± 6 months) (*P* = 0.03).

Physical examination was normal in 19 dogs. Twelve dogs had concurrent medical conditions including heart disease (*n* = 2), hyperadrenocorticism (*n* = 2), renal disease (*n* = 2), hypertension (*n* = 1), liver disease (*n* = 1), macrothrombocytopenia (*n* = 1), polyarthritis (*n* = 1), protein-losing enteropathy (*n* = 1), and syringomyelia (*n* = 1).

The most prominent neurological deficits were ataxia with and without hypermetria, head tilt, nystagmus, decreased menace response and postural reaction deficits. The distribution of neurological signs in relation to infarct topography is described in Table 1.

**Table 1:** Neurological signs in relation extensive vs. limited occlusion of the rostral cerebellar artery in 23 dogs with cerebellar ischaemic stroke

Neurological signs	Extensive RCeA occlusion	Limited RCeA occlusion
Mentation (*n* = 3)		
Obtunded (*n* = 2)		2
Depressed (*n* = 1)		1
Posture and body position		
Head tilt (*n* = 13)		
Contralateral (*n* = 12)	3	9
Unclassified (*n* = 1)		1
Tremors (*n* = 3)		
Intention tremors of head (*n* = 2)	1	1
Unclassified (*n* = 1)		1
Increased tone of limbs (*n* = 2)		
Ipsilateral (*n* = 1)		1
Thoracic limbs (*n* = 1)		1
Decerebellate posture (*n* = 1)		1
Opisthotonus (*n* = 1)		1
Torticollis, contralateral (*n* = 1)		1
Gait abnormalities		
Ataxia (*n* = 21)		
Ataxia with hypermetria (*n* = 11)	1	10
Ataxia without hypermetria (*n* = 4)	1	3
Non-ambulatory (*n* = 6)	2	4
Paresis (*n* = 1)		
Non-ambulatory tetraparesis (*n* = 1)		1
Cranial nerve deficits		
Decreased menace response (*n* = 7)		
Ipsilateral (*n* = 5)		5
Bilateral (*n* = 2)	1	1
Anisocoria (*n* = 3)		
Contralateral mydriasis (*n* = 2)	1	1
Miosis (*n* = 1)		1
Nystagmus, positional (*n* = 8)		
Vertical (*n* = 4)	1	3
Horizontal (*n* = 2)	1	1
Rotatory (*n* = 2)	1	1
Strabismus (*n* = 3)		
Positional (*n* = 2)		2
Spontaneous (*n* = 1)	1	
Postural reaction deficits other than proprioceptive deficits (*n* = 7)		
Ipsilateral (*n* = 5)		5
Bilateral (*n* = 1)		1
Unclassified (*n* = 1)		1
Proprioceptive deficits (*n* = 5)		
Ipsilateral (*n* = 2)		2
Contralateral (*n* = 1)		1
Pelvic limbs (*n* = 1)		1
Unclassified (*n* = 1)		1
Other signs		
Signs of nausea (*n* = 4)		
Vomiting (*n* = 2)	1	1
Salivating (*n* = 2)		2

*RCeA* rostral cerebellar artery. Occlusions were classified as extensive when affecting the entire vascular territory of the rostral cerebellar artery (RCeA) and as limited when affecting a smaller cortical part of the vascular territory of either the RCeA or a perforating artery

Five dogs had a history of a previous episode (between 2 weeks and 3 years prior to stroke event) of sudden neurological deficits resolving within 24 h suggestive of a possible transient ischaemic attack (TIA) [27].

**Fig. 1:**
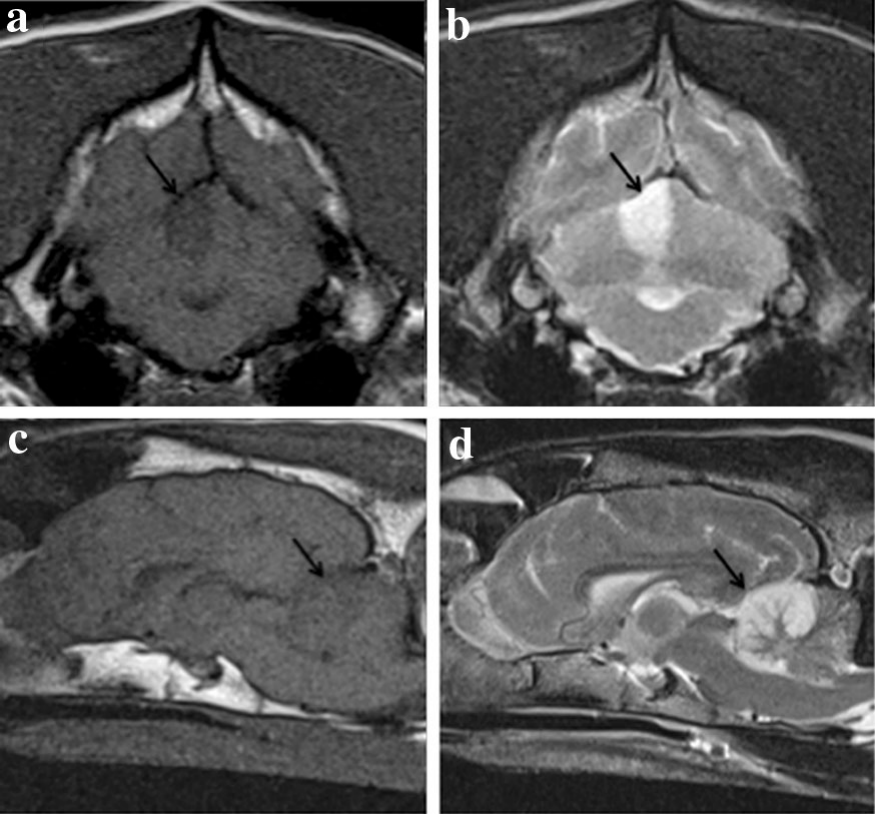
Territorial infarct classified as limited. Brain magnetic resonance imaging (MRI) from a 10-year-old female neutered Lurcher with an acute right-sided rostral cerebellar artery territorial infarct. *Arrows* indicate the cerebellar region affected by the ischaemic stroke. **a** Transverse plane T_1_-weighted (T1W) at the level of the rostral cerebellum, **b** transverse plane T_2_-weighted (T2W) at the level of the rostral cerebellum, **c** mid sagittal plane T1W, **d** mid sagittal plane T2W Images obtained by a 1.5T MRI unit (MAGNETOM Symphony, Siemens Healthcare)

**Fig. 2:**
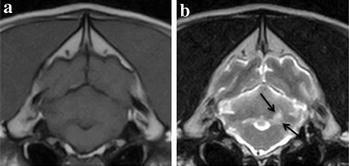
Lacunar infarct classified as limited. Brain magnetic resonance imaging (MRI) of an 8-year-old female neutered labrador retriever obtained within 48 h after onset of neurological deficits. *Arrows* indicate the cerebellar region affected by the ischaemic stroke. **a** Transverse plane T_1_-weighted at the level of the rostral cerebellum, **b** transverse plane T_2_-weighted at the level of the rostral cerebellum Images obtained by a 0.4T MRI unit (Aperto Permanent Magnet, Hitachi)

The median time from onset of clinical signs to MR exam was 1 day. In all dogs, a single cerebellar infarct was identified. All infarcts were located to the rostral and tentorial part of the cerebellum in the territory of the RCeA (right *n* = 10, left *n* = 11, midline *n* = 2), with 19/23 infarcts being territorial and wedge-shaped and 4/23 infarcts being lacunar with a suspected occlusion of a deep penetrating artery from the RCeA resulting in ovoid lesions of the central part of the cerebellum. No significant mass effect or contrast enhancement was seen with any of the lesions (Figs. 1, 2). In addition to conventional MRI, gradient echo imaging, diffusion weighted imaging, and magnetic resonance angiography were performed in 13, 3, and 1 dog, respectively. The lesion appeared hyperintense on gradient echo and diffusion weighted images, and angiography revealed no signs of occlusive disease or vascular malformations. CSF analysis was performed in 10 dogs with unremarkable findings.

**Fig. 3:**
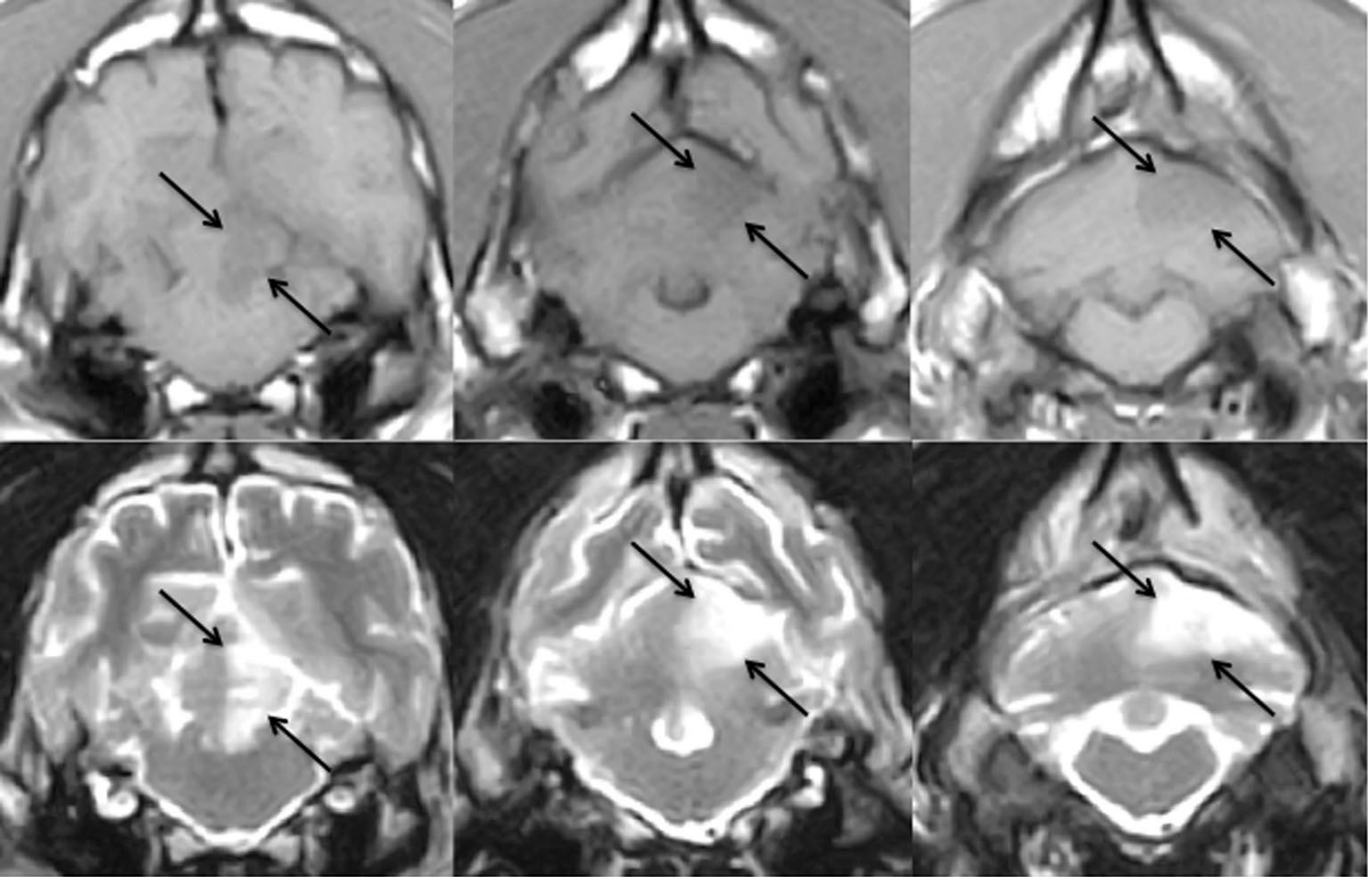
Territorial infarct classified as extensive. Sequential brain magnetic resonance imaging (MRI) from a 9-year-old female neutered English cocker spaniel with an acute right-sided rostral cerebellar artery territorial infarct. Direction of images: rostral to caudal. *First row*: transverse plane T1-weighted images. *Second row*: transverse plane T2-weighted images. *Arrows* indicate the cerebellar region affected by the ischaemic stroke Images obtained by a 0.4 T MRI unit (Aperto Permanent Magnet, Hitachi)

**Fig. 4:**
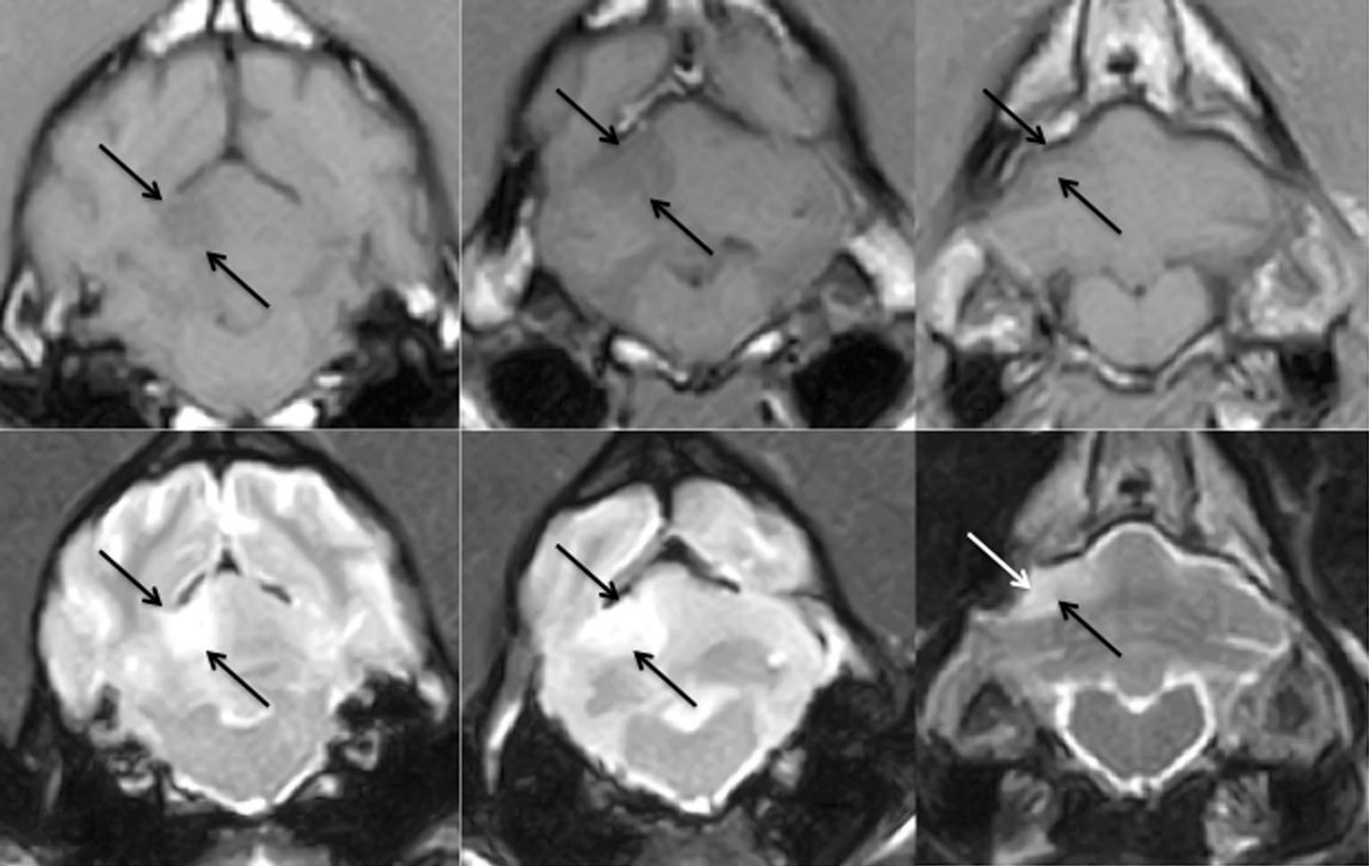
Territorial infarct classified as limited. Sequential brain magnetic resonance imaging (MRI) from an 11-year-old female neutered mixed-breed with an acute left-sided rostral cerebellar artery territorial infarct. Direction of images: rostral to caudal. *First row*: transverse plane T1-weighted images. *Second row*: transverse plane T2-weighted images. *Arrows* indicate the cerebellar region affected by the ischaemic stroke Images obtained by a 0.4T MRI unit (Aperto Permanent Magnet, Hitachi)

All included dogs survived and were discharged within 1–10 days of hospitalisation (median = 1 day). In dogs (*n* = 4) with an extensive occlusion of the RCeA (Fig. 3) the median hospitalisation time was 6.5 days, and in dogs (*n* = 20) with a limited occlusion of the RCeA or a lacunar infarction (Fig. 4) median hospitalisation time was 1 day.

## Discussion

The present study describes a cohort of dogs with cerebellar ischaemic stroke, resulting from the thrombotic occlusion of the RCeA or its branches. The clinical presentation was characterised by a peracute to acute onset of non-progressing neurological deficits with an excellent short-term prognosis.

In accordance with previous studies, the most common sign in affected dogs was ataxia [10, 14, 16]. In humans, the superior cerebellar artery (SCA) corresponds to the RCeA in dogs. The frequent finding of ataxia is in accordance with human studies of infarction of the area supplied by the SCA [28–30]. In the present study, ataxia was commonly accompanied by hypermetria, a sign attributed to uninhibited flexor muscle contraction, which is pathognomonic for a cerebellar or spinocerebellar tract damage [22, 23].

The second most common sign in the dogs was head tilt, a sign that has previously been associated with rostral cerebellar ischaemic stroke [7, 10, 14–16, 18]. In the majority of cases the head tilt was opposite to the lesion representing a so-called paradoxical vestibular syndrome that can be seen with lesions involving the caudal cerebellar peduncle or flocculonodular lobe responsible for the vestibular component of the cerebellum. Damage to these structures and to the fastigial nucleus was presumably also responsible for the nystagmus seen [22, 23]. As the RCeA does not supply these structures [1], this finding is unexpected. Nystagmus has previously been documented to be a frequent sign in dogs affected by rostral cerebellar infarcts [7, 10, 14, 18]. In a study of 293 human patients with cerebellar infarction, nystagmus was also a common finding irrespective of the vascular territory affected [30], and a recent study further supports the finding that infarcts in the territory of the SCA can cause vestibular signs such as nystagmus [31]. In the present study, the vestibular dysfunction was further reflected by the observed strabismus [32], which has previously been reported in both dogs [14, 18] and humans with cerebellar infarction [33].

Regarding the finding of proprioceptive deficits and paresis in some of the dogs, the most likely aetiology is compression of the brainstem secondary to cerebellar oedema formation [7, 26, 34, 35], but as the SCA has collateral branches to the pons and medulla oblongata [36], it is also possible that the observed proprioceptive deficits and paresis were caused by an infarct in one of these areas that was not visible on MRI, as previously suggested [7]. Paresis has been reported in several other studies of dogs with rostral cerebellar ischaemic stroke [7, 14, 16], and in addition a few studies have described the finding of proprioceptive deficits [14, 18]. Likewise, cerebellar damage as a cause of active proprioceptive deficits in humans was recently documented [37].

One dog in the study appeared depressed, which could possibly be a sign of discomfort or pain and a few dogs of the present study appeared obtunded, which may be due to brain stem compression secondary to cerebellar oedema. A few veterinary studies have described dogs affected by rostral cerebellar infarction as being either restless, dull, depressed, or disoriented [10, 14, 18]. Symptoms in humans with SCA infarction such as vertigo, headache and dysarthria [24, 29, 38] may not be identified in dogs. In human patients, the finding of a decreased level of consciousness has been correlated with a worse outcome possibly due to hydrocephalus and brainstem compression [30, 39, 40]. In recent years, research has demonstrated a possible cerebellar impact on cognition [41].

Finally, one dog presented with contralateral torticollis, a sign that is usually considered a consequence of brainstem involvement. Ipsilateral torticollis has previously been reported in one dog with rostral cerebellar infarction [18]. The cause of the observed sign is not known, but recently neuropathological changes specific to the cerebellum were demonstrated in a human post-mortem case-series study from patients with torticollis/cervical dystonia [42]. Although speculative, it is possible that the cerebellar stroke was the direct cause of the observed torticollis.

No immediate cardinal signs justifying the term “syndrome” were identified. However, as dogs did present with a collection of both cerebellar and vestibular signs, dogs showing such neurological characteristics may have suffered a cerebellar ischaemic stroke, and this should alert the clinician of the importance of further diagnostic investigations.

The present study found no obvious association between infarct size, as visualized on MRI, and the observed neurological deficits, i.e. dogs with extensive infarction of the supply area of the RCeA did not show significantly more neurological deficits than the dogs with limited or lacunar ischaemic strokes. For example, when evaluating for some of the most frequently observed neurological signs, a high proportion of all affected dogs exhibited ataxia and/or decreased menace response regardless of whether the infarcts were classified as extensive or limited. A slightly higher proportion of dogs with extensive infarcts presented with head tilt and/or nystagmus, but this was not considered significant due to the small number of dogs in this group. Despite of this lack of association between infarct size and degree of neurological signs observed, a prolonged time of hospitalisation was needed in dogs with extensive occlusion of the RCeA, suggesting that these dogs were actually more seriously affected. This finding is supported by a human study, documenting that large, compared to small, lesions predict a poorer outcome [35], but further studies are needed to investigate this hypothesis in dogs.

A limitation to this study was the low proportion of dogs with extensive infarcts, and it remains unknown whether a higher number of dogs could demonstrate an association between infarct topography and neurological signs. The human cerebellar stroke syndrome may under certain circumstances be further subclassified into three distinct syndromes that describe the signs seen in relation to infarction of the vascular territories of the three major arteries in humans, i.e. the posterior inferior cerebellar artery, the anterior inferior cerebellar artery, and the SCA [26], yet studies have described the difficulty of clinically assessing which part of cerebellum is affected in human patients with ischaemic stroke [25, 30].

Short-term outcome for the dogs included in the present study appeared to be excellent, as all dogs survived and were discharged within 1–10 days. Being referral cases, the results may, however, be biased towards a better outcome, as more severely affected dogs could have been euthanized at the primary practices. Some human studies have reported that cerebellar ischaemic stroke provides a better prognosis than other stroke subtypes [35, 40, 43]. However, disability and death due to complicating oedema formation, subsequent obstructive hydrocephalus and concomitant infarction of the brainstem are known risks in humans [34, 35], and the latter has also been reported in dogs [7]. In one human study of 30 patients suffering from cerebellar infarction, a case fatality rate of 23 % was reported [28], but the relationship between the specific affected cerebellar artery and associated prognosis in humans remains uncertain. Some studies have described superior vs. inferior infarction to be associated with a poorer outcome [30, 40], while another study of 66 human patients with infarction of the SCA vs. infarction of the inferior cerebellar artery territory found that a severe mass effect was only detected in 7 vs. 30 % of the cases [29]. As the RCeA in dogs corresponds to the SCA in humans, this may explain the observed excellent short-term outcome in the dogs of the present study. Importantly, the topographical location of the cerebellum in dogs is different from humans, where the cerebellum is ventral to the cerebrum, and where the human pons has an enlarged conformation [44] in comparison to dogs, which could explain the lack of oedema formation and consequently a better short-term prognosis for dogs with suspected cerebellar ischaemic stroke.

The reason for the high prevalence of rostral cerebellar ischaemic stroke in dogs remains unknown. It is possible that the vascular anatomy of the cerebellum plays an important role where the RCeA usually originates from the caudal communicating artery, thus receiving blood from the carotid arteries, although, in some dogs it originates from the basilar artery [2, 3]. This may predispose dogs to cardiogenic embolism [4]. Furthermore, it has been speculated if the Chiari-like malformation, which is often found in Cavalier King Charles Spaniels, may cause flow disturbances and predispose affected dogs to rostral cerebellar infarction [4, 8]. The canine cerebellar vascularization contrasts with the cerebellar vascularization of humans, where the SCA originates from the vertebrobasilar system [26], even though the cerebellar vasculature can also be an object of individual variation [45]. Interestingly, a recent study of human patients undergoing carotid artery stenting documented that emboli originating from the carotid arteries in a smaller percentage of the patients did actually reach the posterior circulation causing cerebellar infarcts in the territory of the SCA [46].

All dogs in this report had a cerebellar infarction in the vascular area of the RCeA. This was not surprising, as infarcts of the CCeA territory in dogs seem rare. So far, very few dogs with suspected caudal cerebellar infarction have been described. Neurological signs such as depression, head tilt, generalized ataxia, and unilateral postural reaction deficits were reported, as was also the case in the present study. Dogs with CCeA infarction additionally experienced disorientation, rolling episodes, and hyperextended forelimbs [10, 21]. It is likely that there is an anatomical explanation for the low prevalence of caudal cerebellar infarction, as the CCeA originates from the vertebrobasilar system unlike the RCeA that originates from the circle of Willis [1–3]. Another explanation could be that caudal cerebellar ischaemic stroke in dogs may cause either very mild neurological signs that may go by unnoticed, or they may on the contrary cause very serious neurological deficits resulting in euthanasia, in either case preventing the dogs from being referred for further evaluation.

Similar to the findings from previous studies on canine ischaemic stroke [7, 8], 22 % of the dogs investigated in the present study had previously had short neurological episodes prior to the stroke event suggesting a possible TIA. A TIA is characterised by a short episode of neurological dysfunction, commonly lasting less than 60 min, which is caused by transient focal brain ischaemia, with no evidence of subsequent infarction when investigated by MRI [27]. In a study of 2416 human patients with ischaemic stroke, 23 % of the patients had suffered previous TIA [47]. The findings of the present study thus support the hypothesis that dogs can have episodes of TIA and that such episodes may precede a true stroke.

The present study was subject to certain limitations, e.g. a small population and short follow up. As all dogs recovered from the neurological incidence, results from histopathological evaluations were not available. Unfortunately, MRI does not provide a final diagnosis and previous studies have demonstrated the difficulties of differentiating ischaemic stroke from inflammatory diseases and neoplasia [12, 48]. Finally, given the retrospective data collection, MRI examinations of the included dogs did not follow a standardized MRI protocol, which impeded a detailed description of infarct topography including affection of specific arterial branches of the RCeA and the occurrence of haemorrhagic transformation and due to the relatively low number of cases, statistical analyses were not performed.

## Conclusions

Rostral cerebellar ischaemic stroke should be included in the list of possible differential diagnoses in dogs presenting with peracute to acute onset of neurological signs predominated by ataxia and vestibular signs, including contralateral head tilt and nystagmus. Supplementary findings may include mental changes, paresis, proprioceptive deficits, and torticollis, of which paresis and proprioceptive deficits are the most common. The short-term prognosis is excellent, and this encouraging information should be used by clinicians when guiding owners of affected dogs..

## Abbreviations

CCeA: caudal cerebellar artery
MRI: magnetic resonance imaging
RCeA: rostral cerebellar artery
SCA: superior cerebellar artery
T1W: T1-weighted
T2W: T2-weighted
TIA: transient ischaemic attack

## References

[1] Anderson WD, Kubicek W (1971). The vertebral-basilar system of dog in relation to man and other mammals. Am J Anat.

[2] Gillilan LA (1976). Extra- and intra-cranial blood supply to brains of dog and cat. Am J Anat.

[3] DeVos NR, Simeons PJ, Schaller O (2007). Angiologia—arteria. Illustrated veterinary anatomical nomenclature.

[4] Garosi L, McConnell JE, Platt SR, Barone G, Baron JC, de Lahunta A (2005). Results of diagnostic investigations and long-term outcome of 33 dogs with brain infarction (2000–2004). J Vet Intern Med.

[5] Hossmann KA (2006). Pathophysiology and therapy of experimental stroke. Cell Mol Neurobiol.

[6] Gredal H, Toft N, Westrup U, Motta L, Gideon P, Arlien-Soborg P (2013). Survival and clinical outcome of dogs with ischaemic stroke. Vet J.

[7] Garosi L, McConnell JF, Platt SR, Barone G, Baron JC, de Lahunta A (2006). Clinical and topographic magnetic resonance characteristics of suspected brain infarction in 40 dogs. J Vet Intern Med.

[8] McConnell JF, Garosi L, Platt SR (2005). Magnetic resonance imaging findings of presumed cerebellar cerebrovascular accident in 12 dogs. Vet Radiol Ultrasound.

[9] Kent M, Glass EN, Haley AC, March P, Rozanski EA, Galban EM (2014). Ischemic stroke in Greyhounds: 21 cases (2007–2013). J Am Vet Med Assoc.

[10] Gredal H, Skerritt GC, Gideon P, Arlien-Soeborg P, Berendt M (2013). Spontaneous ischaemic stroke in dogs: clinical topographic similarities to humans. Acta Neurol Scand.

[11] Goncalves R, Carrera I, Garosi L, Smith PM, FraserMcConnell J, Penderis J (2011). Clinical and topographic magnetic resonance imaging characteristics of suspected thalamic infarcts in 16 dogs. Vet J..

[12] Cervera V, Wilfried M, Vite CH, Johnson V, Dayrell-Hart B, Seiler GS (2011). Comparative magnetic resonance imaging findings between gliomas and presumed cerebrovascular accidents in dogs. Vet Radiol Ultrasound.

[13] Joseph RJ, Greenlee PG, Carrilo JM, Kay WJ (1988). Canine cerebrovascular disease: clinical and pathological findings in 17 cases. J Am Anim Hosp Assoc.

[14] Irwin JC, Dewey CW, Stefanacci JD (2007). Suspected cerebellar infarcts in 4 dogs. J Vet Emerg Crit Care.

[15] Cook LB, Coates JR, Dewey CW, Gordon S, Miller MW, Bahr A (2005). Vascular encephalopathy associated with bacterial endocarditis in four dogs. J Am Anim Hosp Assoc.

[16] Paul A, Lenard Z, Mansfield C (2010). Computed tomography diagnosis of eight dogs with brain infarction. Aust Vet J.

[17] Thomas WB, Sorjonen DC, Scheuler RO, Kornegay JN (1996). Magnetic resonance imaging of brain infarction in seven dogs. Vet Radiol Ultrasound.

[18] Berg JM, Joseph RJ (2003). Cerebellar infarcts in two dogs diagnosed with magnetic resonance imaging. J Am Anim Hosp Assoc.

[19] Major AC, Caine A, Rodriguez SB, Cherubini GB (2012). Imaging diagnosis—magnetic resonance imaging findings in a dog with sequential brain infarction. Vet Radiol Ultrasound.

[20] Bagley RS, Anderson WI, de Lahunta A, Kallfelz FA, Bowersox TS (1988). Cerebellar infarction caused by arterial thrombosis in a dog. J Am Vet Med Assoc.

[21] Negrin A, Gaitero L, Anor S (2009). Presumptive caudal cerebellar artery infarct in a dog: clinical and MRI findings. J Small Anim Pract.

[22] Holliday TA (1979). Clinical signs of acute and chronic experimental lesions of the cerebellum. Vet Sci Commun.

[23] de Lahunta A, Glass E (2009). Veterinary neuroanatomy and clinical neurology.

[24] Warlow C, van Gijn J, Dennis M, Wardlaw JM, Bamford J, Hankey G (2008). Which arterial territory is involved? Stroke practical management.

[25] Edlow JA, Newman-Toker DE, Savitz SI (2008). Diagnosis and initial management of cerebellar infarction. Lancet Neurol.

[26] Lee H (2009). Neuro-otological aspects of cerebellar stroke syndrome. J Clin Neurol.

[27] Albers GW, Caplan LR, Easton JD, Fayad PB, Mohr JP, Saver JL, TIA Working Group (2002). Transient ischemic attack—proposal for a new definition. N Engl J Med.

[28] Macdonell RA, Kalnins RM, Donnan GA (1987). Cerebellar infarction: natural history, prognosis, and pathology. Stroke.

[29] Kase CS, Norrving B, Levine SR, Babikian VL, Chodosh EH, Wolf PA (1993). Cerebellar infarction. Clinical and anatomic observations in 66 cases. Stroke.

[30] Tohgi H, Takahashi S, Chiba K, Hirata Y (1993). Cerebellar infarction. Clinical and neuroimaging analysis in 293 patients. The Tohoku cerebellar infarction study group. Stroke.

[31] Lee H, Kim HA (2013). Nystagmus in SCA territory cerebellar infarction: pattern and a possible mechanism. J Neurol Neurosurg Psychiatry.

[32] de Lahunta A, Glass E (2009). Vestibular system: special proprioception. Veterinary neuroanatomy and clinical neurology.

[33] Rowe F, VIS group UK (2010). The profile of strabismus in stroke survivors. Eye..

[34] Cano LM, Cardona P, Quesada H, Mora P, Rubio F (2012). Cerebellar infarction: prognosis and complications of vascular territories. Neurologia.

[35] Ng ZX, Yang WR, Seet E, Koh KM, Teo KJ, Low SW (2015). Cerebellar strokes: a clinical outcome review of 79 cases. Singapore Med J.

[36] Nanda BS, Getty R (1975). Blood supply to the brain. Sisson and Grossman’s the anatomy of domestic animals.

[37] Bhanpuri NH, Okamura AM, Bastian AJ (2013). Predictive modeling by the cerebellum improves proprioception. J Neurosci.

[38] Datar S, Rabinstein AA (2014). Cerebellar infarction. Neurol Clin.

[39] Jauss M, Krieger D, Hornig C, Schramm J, Busse O (1999). Surgical and medical management of patients with massive cerebellar infarctions: results of the German–Austrian cerebellar infarction study. J Neurol.

[40] Kelly PJ, Stein J, Shafqat S, Eskey C, Doherty D, Chang Y (2001). Functional recovery after rehabilitation for cerebellar stroke. Stroke.

[41] Buckner RL (2013). The cerebellum and cognitive function: 25 years of insight from anatomy and neuroimaging. Neuron.

[42] Prudente CN, Pardo CA, Xiao J, Hanfelt J, Hess EJ, Ledoux MS (2013). Neuropathology of cervical dystonia. Exp Neurol.

[43] Ng YS, Stein J, Ning M, Black-Schaffer RM (2007). Comparison of clinical characteristics and functional outcomes of ischemic stroke in different vascular territories. Stroke.

[44] Felten DL, Shetty AN (2010). Section II: regional neuroscience brain Stem and cerebellum. Netter’s atlas of neuroscience.

[45] Marinkovic S, Kovacevic M, Gibo H, Milisavljevic M, Bumbasirevic L (1995). The anatomical basis for the cerebellar infarcts. Surg Neurol.

[46] Zhu L, Wintermark M, Saloner D, Fandel M, Pan XM, Rapp JH (2011). The distribution and size of ischemic lesions after carotid artery angioplasty and stenting: evidence for microembolization to terminal arteries. J Vasc Surg.

[47] Rothwell PM, Warlow CP (2005). Timing of TIAs preceding stroke: time window for prevention is very short. Neurology.

[48] Young BD, Fosgate GT, Holmes SP, Wolff CA, Chen-Allen AV, Kent M (2014). Evaluation of standard magnetic resonance characteristics used to differentiate neoplastic, inflammatory, and vascular brain lesions in dogs. Vet Radiol Ultrasound.

